# Targeting Differential Roles of Tumor Necrosis Factor Receptors as a Therapeutic Strategy for Glaucoma

**DOI:** 10.3389/fimmu.2022.857812

**Published:** 2022-05-16

**Authors:** Lidawani Lambuk, Suhana Ahmad, Muhammad Zulfiqah Sadikan, Nor Asyikin Nordin, Ramlah Kadir, Nurul Alimah Abdul Nasir, Xin Chen, Jennifer Boer, Magdalena Plebanski, Rohimah Mohamud

**Affiliations:** ^1^Department of Immunology, School of Medical Sciences, Universiti Sains Malaysia, Kota Bharu, Malaysia; ^2^Centre for Neuroscience Research (NeuRon), Faculty of Medicine, Universiti Teknologi MARA, Sungai Buloh, Malaysia; ^3^State Key Laboratory of Quality Research in Chinese Medicine, Institute of Chinese Medical Sciences, University of Macau, Macau SAR, China; ^4^School of Health and Biomedical Sciences, Royal Melbourne Institute Technology (RMIT) University, Bundoora, VIC, Australia

**Keywords:** tumour necrosis factor, TNFR1, TNFR2, glaucoma, retinal ganglion cells, neurodegeneration, neuroprotection, neuroinflammation

## Abstract

Glaucoma is an irreversible sight-threatening disorder primarily due to elevated intraocular pressure (IOP), leading to retinal ganglion cell (RGC) death by apoptosis with subsequent loss of optic nerve fibers. A considerable amount of empirical evidence has shown the significant association between tumor necrosis factor cytokine (TNF; TNFα) and glaucoma; however, the exact role of TNF in glaucoma progression remains unclear. Total inhibition of TNF against its receptors can cause side effects, although this is not the case when using selective inhibitors. In addition, TNF exerts its antithetic roles *via* stimulation of two receptors, TNF receptor I (TNFR1) and TNF receptor II (TNFR2). The pro-inflammatory responses and proapoptotic signaling pathways predominantly mediated through TNFR1, while neuroprotective and anti-apoptotic signals induced by TNFR2. In this review, we attempt to discuss the involvement of TNF receptors (TNFRs) and their signaling pathway in ocular tissues with focus on RGC and glial cells in glaucoma. This review also outlines the potential application TNFRs agonist and/or antagonists as neuroprotective strategy from a therapeutic standpoint. Taken together, a better understanding of the function of TNFRs may lead to the development of a treatment for glaucoma.

## Introduction

Glaucoma, an ocular neurodegenerative disease, is a leading cause of irreversible blindness that has affected over 70 million people worldwide (1). The hallmark feature of glaucoma is axonal (optic nerve) degeneration with progressive loss of retinal ganglion cells (RGCs) somas (2). Currently, glaucoma management predominantly aims to lower the intraocular pressure (IOP) as it is considered the primary risk factor for the initiation and progression of glaucomatous optic neuropathy (3). Yet, a significant number of glaucoma cases continuously exhibit vision loss even when the IOP is controlled, and most IOP-dependent medications are accompanied by adverse effects (1). Therefore, there remains a need for a novel therapeutic approach that can provide neuroprotection to slow or rather prevent progressive loss, which can preserve RGCs survival and visual function (4). The key to successful therapeutic intervention is to understand the underlying mechanisms caused by various stresses leading to optic neuropathy (5).

The axonal damage caused by injury to the optic nerve is thought to be the first stage of insult in glaucoma. However, direct impact on the optic nerve may ultimately result in RGCs death, which is exacerbated by the production of inflammatory cytokines from resident innate immune cells (glia) and the complement system in retina (6). Meanwhile, the second stage of degeneration concept in glaucoma is associated with the activated glia, which produces cytotoxic molecules such as the inflammatory cytokines that can severely damage the surviving RGCs (7). Although the mechanisms causing RGC degeneration in glaucoma remain obscure, a strong relationship between molecularly defined apoptosis and inflammation has been revealed, indicating that inflammation contributes to the demise of RGCs (1, 8).

Recently, a plethora of literature suggests neuroinflammation as one of the significant contributors to RGC loss in glaucoma. One of the key players could be tumour necrosis factor alpha (TNF; TNFα) (9, 10). Since it was first discovered cytotoxic to tumour cells and primary inducer of tumour regression in the past, TNF in recent times is perceived as the potent mediator in apoptosis (11, 12). This suggests the apparent importance of TNF in the pathogenesis of human diseases. Amongst 19 family members of TNF superfamily (e.g., TNFβ [also known as lymphotoxin alpha], lymphotoxin beta, FasL, TNF-related apoptosis-inducing ligand CD27L, CD30L, and CD40L), TNF is viewed as the most prominent inducer of apoptosis and regulator of immune response in healthy as well as in disease organisms (13). The physiological antithetic action of TNFRs occurs *via* TNF receptor I (TNFR1) and TNF receptor II (TNFR2). Various cellular responses can elicit TNF expressions through these receptors. It depends on many factors, including the metabolic state and the adaptor proteins present in the cells (14). The deregulation in the activation of TNFR1 and TNFR2 could shift the harmonious biological effects of TNF physiological activity, hence triggering cells death and tissue degeneration.

Divergent actions of TNF in neurodegenerative diseases have been long implicated. Because of the opposite natures, we reasoned that both an inhibition of TNFR1 signaling and selective activation of TNFR2 signaling, respectively, could shift the balance of endogenous TNF activity toward an overall neuroprotective/regenerative response in retinal neurons (15). Since TNF is known as the dominant mediator in pro-inflammatory functions of the central nervous system (CNS), it is imperative to understand the differential roles of TNFRs in glaucomatous neurodegeneration. Although TNF-associated glaucoma has been well documented, its receptors are severely understudied. Throughout literature, there is currently a lack of consensus on the functional outcomes of TNF/TNFRs signaling in glaucoma, resulting in conflicting reports and suggesting a dire need to investigate the cellular and molecular mechanisms that govern TNF pathways. The authors attempt to discuss the differential roles of TNFR1 and TNFR2 in glaucomatous conditions. This review also briefly discusses the potential application of TNF agonists and/or antagonists, which may facilitate the safe translation of basic research findings from animal to human. This is the first review article focusing on glaucoma in relation to the associations of TNF and its receptors. In align with Chen et al. (16), we suggest that the regulation of TNFR2, particularly expressed on regulatory T cells (Treg) is crucial in maintaining tolerance and immune homeostasis (16–18). However, in this review, we would like to suggest the current development of anti-TNF therapies aimed at selectively inhibiting deleterious effects of this cytokine while maintaining its physiological functions *via* these two receptors. Collectively, a better understanding of the differential roles of TNFRs may lead to the development of a treatment for glaucoma.

## Ocular Immune Surveillance in Glaucoma

Central nervous system (CNS) is considered to be an immune-privileged system. It comprises a network of vessels with a very specialized endothelial lining supported by macroglia, microglial, pericytes, and neurons. Together these cells form a substantial blood-brain barrier (BBB). The latter strictly regulates the immune cells entering the CNS and maintains homeostasis concomitantly. However, under inflammatory conditions, the BBB gets disrupted, and immune cells migrate into the CNS parenchyma. Retinal neurons, including RGCs, are part of the CNS. They share several similar properties with other CNS neurons, including the inability to regenerate in response to cellular damage. The immune-mediated inflammation in the retina is tightly regulated by the specialized intraocular environment with tight interconnected junctions in a similar fashion to BBB, referred to as the blood-retinal barrier (BRB) (19). This barrier makeup of endothelial cells of the inner retinal vasculature (inner BRB) and retinal pigment epithelial cells (outer BRB) confer a stringent permeation of cells or macromolecules from the circulation into the retina that requires active transport to cross the barrier (20). Although showing many similarities, some essential differences between the BRB and BBB have recently been documented (21). This also includes the expression of tight junction-associated genes was reported to be more strongly expressed in the BBB than in the inner BRB (21). In addition to that, the BBB is established by endothelial cells rather than by epithelial cells for outer BRB, which explains the former requires the influence from the proximal astrocytes to activate and maintain its barrier function (22). Unlike BBB and inner BRB, the retinal pigment epithelial (RPE) cells within the outer BRB can provide barrier characteristics in the absence of astrocytes, enabling a high degree of control of fluid permeability. The pericyte–endothelial cell ratio in the developing retina is generally higher than in the brain, suggesting a more significant requirement for pericytes in the retina to maintain barrier integrity than the brain (23). For specific functions and characteristics of BRB, refer to reviews (20, 23, 24).

According to Streilein, the ocular immune-privileged is attributed to the immunosuppressive and anti-inflammatory properties of the ocular cells, tissues, and fluids (25). For instance, the blood-aqueous barrier and absence of a lymph drainage system but highly vascularized with residents of immune cells in the uvea such as macrophages and dendritic cells, have been revealed to prevent extensive damage against intraocular inflammation (26) and help to minimize the risk of vision integrity (27). Because of their proximity to retinal neurons, resident glial cells, comprised mainly macroglia (i.e., astrocytes and Müller cells) and microglia, perform vital tasks in the normal physiological condition of the retina. As such, glaucoma is considered a neuroinflammatory disease since it is defined by dying RGCs accompanied by the active resident glial cells and infiltration of circulating immune cells (28, 29). In a physiological state, these glial cells preserve the healthy state of the retina but turn into innate immune cells upon following injury (30–32). They act as antigen presenting cells and secrete molecules including potent cytokines and neurotrophic factors into the retina to repair the damage and expel critically injured neurons (33). However, prolonged activation of glial cells has been suggested to dampen endogenous mechanisms for RGC survival and damage with more significant neuronal loss. Indeed, the overactivation of glial cells has been one of the fundamental concepts in the mechanism of RGC loss (34–36). In contrast to the latter paradigm, some studies suggest that the innate immune response is critical in the RGC defense system after injury (37, 38).

Recent studies have focused on immunological changes occurring in glaucoma pathogenesis, and potential preventative therapies based along those lines have been proposed. The major targets of interest are cytokines; as in the aforementioned, the inflammatory mediators play an essential role in the early progression of glaucoma and may regulate RGC survival or death (39, 40). In such event, inflammation occurs when triggered by primary injury accompanied by the active release of reactive oxygen species, glutamate, nitric oxide, and cytokines, including TNF, by the macroglia or microglia (41). Of note, those of old age associated with optic neuropathy are at risk of developing glaucoma. This element reduces the cellular viability of retinal cells and the capability to renew themselves, thereby priming dysfunctional microglia. Such age-related attrition led to an exacerbated microglial response to inflammatory stimuli and may contribute to failure in glial supportive functions (42). The increasing incidence of age-related glaucoma also has been linked to the failure to remove senescent cells (cell-cycle arrest associated with aging) and progressive degenerative changes in trabecular meshwork cells in response to age-related oxidative stress (43). Age is also accompanied by biomechanical changes of the optic nerve (44). Indeed, biomechanical changes in the ONH occur in the aging eye is one of the significant causes of the accelerating progression of glaucoma. Ageing ONH is likely to be associated with stiffness of connective tissues and diminished circulation leading to damage of the axons of RGCs (45).

## General Biology of TNF and Its Receptors

TNF is a multicomplex cytokine that plays as a prime inductor in the modulation of inflammatory cellular events and immune regulation (11). TNF executes its physiological functions *via* the primary members of TNFR superfamily and high-affinity receptors; TNFR1 (p55) and TNFR2 (p75), that are expressed on different cell types and secreted predominantly by various inflammatory cells (46). Traditionally, TNF exerts its pro-inflammatory functions *via* TNFR1 alone and *via* a cooperation between TNFR1-TNFR2 (47). Altogether, TNFR1 is essential to induce pro-inflammatory TNF-α responses, while TNFR2 may primarily mediate cell activation, migration, or proliferation (48). The expression of TNFR2 is at a low level and restricted to specific cell types, including immune cells (e.g., Treg, monocytes and microglia), endothelial cells, neurons, and oligodendrocytes (48). Although it is well known that the TNF is primarily secreted by reactivated glial cells (49), the localized expression of its receptors in the retina and the mechanisms underlying its effects are still under investigation. It is unclear whether the bioactivities of the TNFRs are exerted directly by signaling in RGCs and other retinal neurons or indirectly by numbers of glial and non-neural cells. *In situ* hybridization was used to detect both TNFR1 and TNFR2 in the corneal endothelium, iris, ciliary body, choroid, optic nerve sheath, and vitreoretinal interface in normal mice eyes, however only TNFR2 was detected in the RGC layer (50). Whereas in normal human tissues, the cornea, iris pigment epithelial cells, the ciliary body, vitreous body, and retina express both TNFR1 and TNFR2 (51–53). The vast majority of TNFR-related ocular studies, however, seem to concentrate on TNFR1. A study done by Tezel and colleagues revealed that TNFR1 was predominantly localized in RGCs and TNF cytokines was shown to be more abundant in the inner retinal layers of glaucomatous eyes (54). Further supporting the notion, a study of retinal cell culture suggested that the majority of TNFR1 can be found in nerve bundles located in the anterior region of the glaucomatous ONH, showing that retinal neuronal tissue is an essential target for the effects of TNF that are produced by glial cells (55). This is parallel with the location of retinal glial cells, since astrocytes are typically found in the RGC and nerve fibre layers, whereas Müller cells have their cell bodies in the inner nuclear layer (33). TNFR2 is minimally expressed physiologically and pathologically in the CNS, but it is explicitly upregulated in neurological disease. However, little is known about its localized expression in retinal cells and the current knowledge of TNFR2 in ocular diseases is unfortunately limited. TNFR1 is ubiquitously expressed on various cell types. TNFR1 also efficiently induced pro-inflammatory pathways by interacting with transmembrane homo-trimeric TNF (tmTNF) and soluble homo-trimeric TNF (sTNF) ligands. In the treatment of autoimmune disease models, sTNF inhibitors or TNFR1 antagonists (e.g., XPro1595 and XENP1595 (PEGylated derivative), DMS5540, ATROSAB, TROS and R1antTNF) have been shown to specifically block the sTNF/TNFR1 signal while leaving the TNFR2 signaling pathway intact, indicating a dominating role sTNF towards TNFR1 compared to TNFR2 (48, 56–60). Unlike TNFR1, TNFR2 is exclusively initiated by tmTNF and often causes immune modulation and tissue regeneration (48). Furthermore, tmTNF appears as the main ligand for TNFR2, which has been suggested to be important in local inflammatory responses (61). In Treg, tmTNF plays a critical role in balancing the upregulation or downregulation of Treg immunosuppressive activity, as shown in the deficiency of TNFR2 expressions caused undesirable immune responses in various autoimmune diseases (14). Interestingly, tmTNF within cell may exert “reverse signaling” when interacting with TNFR1/TNFR2 causes the activation of canonical nuclear factor kappa B (NF-κB) or mitogen-activated protein kinase (MAPK)/extracellular signal-regulated kinase (ERK) signaling pathways (62). In contrast to its function as ligand, tmTNF as a receptor may elicit pro-inflammatory effect as demonstrated by Jeucken et al. on its overexpression resulted in enlarged peripheral lymph nodes and spleen (63). Nonetheless, the involvement of tmTNF in immune plasticity warrants further investigation. Altogether, these findings suggest that TNF enhanced expression of adhesion molecules and TNF-induced cytotoxicity are partially abrogated by blockade of either receptor.

The actions of TNFR1 and TNFR2 have been ascribed by their varying expression profiles, ligand binding affinity, and downstream signaling pathway activation, although the majority of the TNF effects are transmitted through TNFR1 (12, 61). TNFR1 contains cytoplasmic death domain (DD) receptors which activate the pro-inflammatory markers and can directly trigger programmed cell death (i.e., apoptosis and necroptosis) (12). The pro-inflammatory effect of TNFR1 is determined by the complex interplay of TNFR1-induced classical NF-κB signaling and apoptosis. Ligand passes to TNFR1 and induces TNFR2 to enhance and regulate TNF effects. Under normal conditions, TNF/TNF-R1 activates NF-κB pathway, which can inhibit the TNF-induced neuronal death process upon injury (54). In most pathophysiological conditions, abnormal TNF production and TNFR1 expression can be detrimental to cell survival or aggravate cell damage (35). Notably, blockage of TNFR1 exhibits pro-survival signaling of TNF in the course of acute inflammation; however, TNFR1 deficiency, not TNFR2, could exacerbate chronic inflammation (64). This could be due to the systemic inflammatory response augmented dramatically and confers protective role in TNFR1 deficiency. Importantly, TNFR1 also generates multiple apoptotic pathways that can integrate and act on mitochondria, leading to reactive oxygen species increase, activation of the pro-death Bcl-2 family members, and c-Jun N-terminal Kinase (JNK) (47). Conversely, the cascading sequence of molecular events of TNFR2 involving the Akt or also known as Protein Kinase B (Akt/PKB) and the alternative NF-κB pathways contribute to eventual neuronal survival (15).

Nonetheless, elucidation of the specific molecular triggers for TNFR2 has proven to be poorly understood. Initially, most information regarding TNF is associated with TNFR1; hence, the TNF-derived TNFR2 signaling is likely underestimated. However, TNFR2 has proved to be far more elusive in its signal transduction pathways in recent years. On the contrary to TNFR1, TNF/TNFR2 interactions do not link to DD-containing adaptors, and their signaling mainly activates the PKB and NF-κB pathways (15). TNFR2 deficiency has been demonstrated to elevate neuronal toxicity, showing the importance of preserving TNFR2 so that it can perform its functions in the neuroprotective pathways (65). Plus, selective inhibition and dysfunction of neuronal TNFR2 enhanced pathological features and diminished microglia activity, which is needed for neuronal clearance (66).

**Figure 1:**
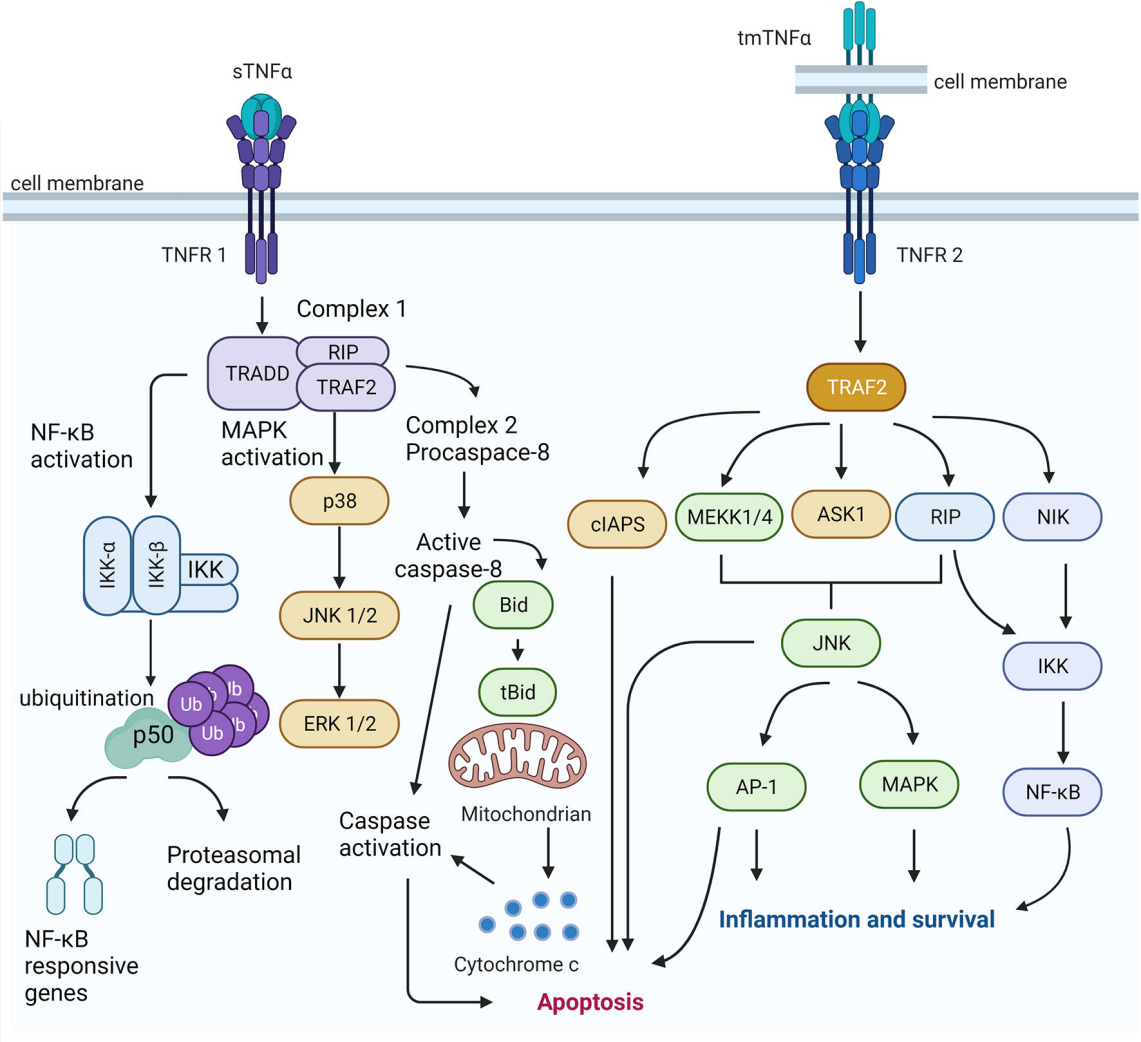
TNFR1 and TNF2 signaling pathways. The sTNFα enhances the TNFR1 induced classical NF-kB signaling and apoptosis. The activation of TNF signaling from complex I result in the activation of the transcription factor of NF-kB-associated responsive genes. It activates different MAPK cascades, which ultimately activates the ERK signaling. On the other hand, TNF-induced signaling can give inhibition of caspase 8, which may trigger an alternative mode of programmed cell death (i.e., necroptosis). The occupancy of TNFR2 by tmTNF leads to the recruitment of TRAF2 and a cellular inhibitor of apoptosis protein-1 (clAP-1) and cellular inhibitor of apoptosis protein-2 (clAP-2). Signaling TRAF2 is also thought to activate a mitogen activated protein kinase kinase kinase (MAPKKK), such as or apoptosis-stimulated kinase 1 (ASK1) or extracellular signal-regulated kinase kinase 1 (MEKK1), in a complex at or near the receptor and Protein kinase RIP, which is the activation of the transcription factor NF-kB is required to the functioning of a third arm of the TNF signaling network. TNFR2 has been suggested to provoke cell proliferation and survival. Created using BioRender (https://biorender.com/).

Overall, TNFR1 and TNFR2 are differently activated and induce signaling of their ligands mainly in the opposite manner. However, they are engaged with proteins and can be interconnected by homogenous regulatory circuits (**Figure 1**). TNFRs signaling constitutes a complex signaling network with synergistic or antagonistic effects depending on the circumstances or stimulants.

## Roles of TNF, TNFR1, and TNFR2 in Glaucomatous Neurodegeneration

**Figure 2:**
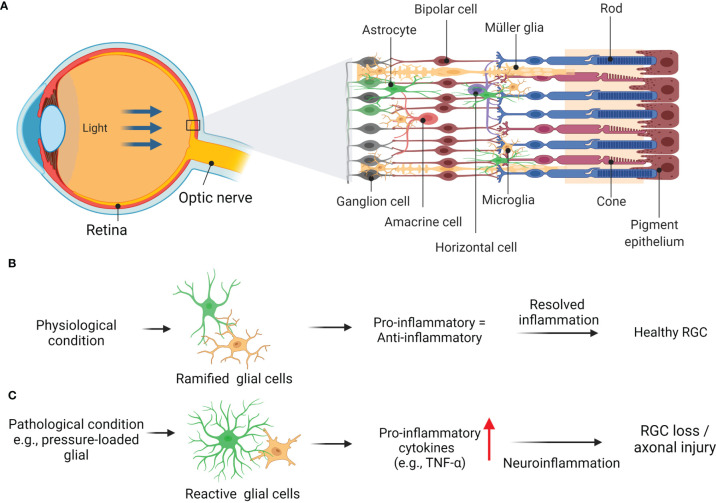
Schematic diagram showing basic anatomy of eye **(A)** with retinal layers and glia-driven inflammation in healthy retina **(B)**. Glaucoma related insult e.g., pressure-loaded glial, generated and expressed by retinal glial cells, promotes neurotoxicity to RGCs and their axonal loss **(C)**. Adapted from “Structure of The Retina” by BioRender.com (2022). Retrieved from https://app.biorender.com/biorender-templates.

Given that TNF is highly generated and expressed by retinal glial cells, this potent pro-inflammatory cytokine plays an essential role in promoting RGC apoptosis in glaucoma (**Figure 2**) (67, 68). For example, up-regulated TNF has been shown to propagate the inflammatory pathways following ischemic or pressure-loaded glial cells, such as inducible nitric oxide synthase is highly secreted by astrocytes, resulting in oligodendrocytes death and enhanced progression of apoptotic RGC (54, 69). It is noteworthy to mention that the TNF cytokines level in the aqueous humour (AH) of individuals with POAG exhibited greater than that of healthy (70, 71), suggesting the potential of TNF as a POAG biomarker. However, AH sampling is an intrusive procedure that is impracticable in clinical settings. Of note, an essential aspect of TNF that should be considered is the relationship between the systemic levels of TNF and the risk of developing or monitoring the progression of glaucoma. Due to its accessibility and potential for screening purposes, blood sampling is an excellent option. This is important as high plasma levels of TNF has been revealed to be associated in patients various racial or ethnic group with POAG and pseudoexfoliation glaucoma (PEG) (67, 67, 72, 73). Adding weight to the idea, a recent study done by Tan and group have demonstrated the potential biomarkers for POAG in both AH and plasma that are correlated with clinical characteristics in patients (74). Since there are no biomarkers available for the use in the clinical setting, it would be interesting to explore in future studies whether increased proinflammatory cytokine levels in blood, including TNF has a significant impact on glaucoma diagnosis, prognosis, and treatment monitoring, particularly in the early progression of the disease.

On the other hand, the TNFR phenotypes’ roles in glaucoma revealed TNF gene polymorphisms that either increase or reduce the risk of glaucoma (75–77). As a pleiotropic cytokine, the regulation and role of TNF in glaucoma are also varied. The role of TNF is further supported when blocking using etanercept is protective against RGCs death, despite the persistent elevation of IOP. Etanercept is a biologic fusion protein between two ligand-binding portions of human TNFR2 fused together with Fc portion of human IgG1 and binds to both sTNF and tmTNF. Recently, it is established that TNFR2 is preferentially expressed on Tregs, a population of cells responsible for immune suppression (16, 78). In glaucoma, Tregs are evidenced to be elevated peripherally in POAG patients compared to healthy controls (79, 80). Inflammatory response resulting from insults (age, steroid, uveitis, alkali injury, surgery) would induce a variety of cell types, including astrocytes, microglia, and peripherally derived immune cells in the optic nerve and/or retina (10). Induction of these cells would initiate a number of inflammatory pathways, including TNF pathway, and drive neuroinflammation in glaucoma. Thus, the elevation of Tregs suggested the presence of persistent inflammation that implicates neurodegeneration etiology in glaucoma.

TNF binds to the low affinity of TNFR1 to activate signaling of pro-inflammatory mediators and elicits caspase-mediated pathway. In normal tissue, TNFR1 is constitutively expressed in the vasculature of ONH and is further up-regulated in astrocytes and glial cells by TNF stimulation observed in glaucomatous degeneration. Furthermore, the specific role of TNF in the induction of RGC death *via* TNFR1 is supported by protection against neuronal loss in TNFR1 knockout mice (35). Also, in the retinal ischemia-reperfusion injury model, neuronal cell survival was more significant in deficient TNFR1 (15). Additionally, a study on unilateral optic nerve crush injury in TNFR1-knockout mice by Tezel et al. (35) reported less glial activation and prominent downregulated expression of TNF (35). This further supports the notion that TNFR1 signaling is responsible for propagating glaucomatous neurodegeneration. On the contrary, in a study using ischemia-reperfusion-induced retinal damage in mice, TNFR1 is revealed to augment neuronal death while TNFR2 promotes neuroprotection (15). Furthermore, the neuroprotective effect of TNFR2 is correlated with the presence of activated Akt/PKB, which is one of the TNFR2-exclusive signaling components, in the reduction of neuronal cell loss in TNFR1 knockout animals. Indeed, activated Akt/PKB can phosphorylate and inhibit pro-apoptotic proteins (81). Likewise, an experiment using 24 hours of TNF treatment reported that neurons with greater phosphorylated Akt/PKB expressions were significantly preserved against glutamate-induced excitotoxic death (82). The neuroprotective actions of TNF through TNFR2 also involve recruitment of TRAF-2 and subsequent activation of NF-κB (83). In contrast to TNFR1, the binding of TNF to the high affinity TNFR2 activates the ubiquitous transcription factor NF-κB, subsequently mediates the gene transcription essential for neuronal survival and promotes neuroprotection (82, 84, 85). The activation of TNFR2 in microglia is suggested to generate anti-inflammatory pathways like those driven by granulocyte colony-stimulating factor, adrenomedullin and interleukin 10 (86). Despite of that, TNFR2 was highly expressed in oligodendrocyte and RGC death induced by TNF in a model primary angle-closure glaucoma (PACG), indicating the equivocal role of TNFR2 signalling in mediating cell survival as proposed by the previously mentioned studies (87). Further strengthen the contradiction, Nakazawa and group observed the detrimental effects of TNF on retinal photoreceptors is mediated through TNFR2, not TNFR1 (88). TNFR2 also might escalate the TNFR1 mediated cell death or inflammation signaling pathways.

In sum, TNF/TNFR1 and/or TNF/TNFR2 interactions in the glaucomatous neurodegeneration seem complex. Elevated serum expression of TNF has been consistently observed in human glaucoma, and its overexpression somewhat shifted towards TNFR1 than TNFR2. Indeed, these observations support the idea that cell survival costs depend on the delicate balance between the two receptors. Any shift in equilibrium might be detrimental, thus promoting neuronal loss. Despite TNF being the subject of interest in ocular studies, investigation of their mechanism involved and their relevance on TNF antagonists in animal model glaucoma have not been thoroughly addressed so far. Conclusively, perhaps an approach exclusively investigating the TNFR2 functions in retinal neuronal and glial activities may gather a better understanding for efficacious neuroprotection in glaucoma.

## TNF Inhibitors

Currently, accumulating evidence supports the ligation of TNF to TNFRs in the glaucoma model, although the therapeutic roles of TNF antagonist mediated RGCs survival have yet to be explored. Other than in glaucoma, TNF is also known to play a significant role in the initiation and modulation of immunity and inflammation in multiple neurodegenerative diseases, including stroke, Alzheimer’s disease (AD), Parkinson’s disease (PD), encephalopathy, meningitis, multiple sclerosis (MS), neuromyelitis optica spectrum disorder (NMOSD), myelin oligodendrocyte glycoprotein (MOG) antibody-associated disease, neuropathy as well as myelosuppression (14, 65, 89, 90). This cytokine with high expressions in the nervous system strongly stimulates demyelination, axonal injury, and enhanced BBB permeability (91). In autoimmune diseases where the immune body mistakenly attacks and destroy healthy cells have been linked to excessive expression of TNF associated with chronic inflammation (92). Therapeutics that specifically modulate the signaling mechanisms of TNF, i.e., blocking TNFR1 actions and/or increasing the TNFR2 signaling pathway, could significantly reduce the side effects of current anti-TNF approaches (93). There have been several TNF blockers, including etanercept, rivastigmine, adalimumab, golimumab, infliximab, and certolizumab, clinically approved to treat autoimmune and neurodegenerative diseases (93).

Interestingly, patients with rheumatoid arthritis (RA) and psoriasis were reported with a low risk of developing AD when treated with TNF blockers therapy (i.e., etanercept, adalimumab, and infliximab) (68), which suggests that blocking TNF expression may have a neuroprotective effect against AD. The same trend was reported in patients with inflammatory bowel disease-associated PD (94). Apart from the neuroprotection and regeneration, TNFR2 has been served in the remyelination of oligodendrocytes (48, 65). Genetic fusion of tenascin C synthesized into a soluble TNFR2 agonist (TNC-scTNFR2) has been established to protect against oxidative stress injury in human differentiated neurons (66). It has been reported that TNFR1 expressions and binding affinity are enhanced while TNFR2 decreases in the brain tissues of AD in humans (65, 95). The intracerebroventricular drug injection of infliximab that blocks against TNF has been shown to suppress amyloid plaques and tau phosphorylation within 3 days, together with improved spatial memory in AD-induced mice (96, 97). Moreover, AD female subjects showed better cognitive function following the intrathecal administration of infliximab (98). Not only that, the cognitive impairment has also improved in a few minutes of peri-spinal administration of etanercept (99).

It has been reported that there is an increased case of demyelinating disorders and opportunistic infections of CNS following TNF blockade. However, neurodegenerative diseases like dementia may also benefit from anti-TNF therapy. One of them is thalidomide, a drug that can reach CNS and block TNF and related cytokines local generation by microglia responsible for neuroinflammation (100). Due to the inefficient permeability to cross BBB, the rationale for applying this therapy is inadequate. Additionally, a study measuring the efficacy of TNF blockers using magnetization transfer ratio histogram peak-heights on grey and white matter indicated parenchymal loss of integrity secondary to TNF blockers in RA and psoriatic disease were not linked with neurocognitive weakening (101). When the target of the TNF blockers falls within both CNS and peripheral nervous system, its efficacy is limited to the complex and invasive route of delivery. As far as patient compliance is concerned, orally administered drugs like thalidomide and its analogs are the recent focus. However, the intake may have disrupted the early TNF production and neither the released protein nor the receptor level. From that perspective, potential protective effects of thalidomide analogs might be useful against neurodegenerative disorders by elucidating the mechanism of action and actual functions of TNF blockers in both acute such as stroke, head trauma, and chronic states (e.g., AD, PD, and amyotrophic lateral sclerosis). This could be due to the TNF blockers that were shown to possess positive outcomes in the pilot study of AD. Since it was expected that there would be more than 13 million AD patients in the United States, this new therapeutic approach is very crucial in terms of new targets and other common life-threatening neurological diseases.

## Potential Therapy of TNF/TNFR in Glaucoma

Since neuroinflammation can lead to neurodegeneration, protecting the neurons from neurotoxicity and stimulating endogenous recovery is necessary. Given that neuroinflammation affects different neuronal sites, immunomodulation seems to be a potential strategy to protect RGC somas, synapses, and axons during neurodegeneration in glaucoma (2). Glial cells are thought to be the best target to recover immune homeostasis and reduce the effects of glaucoma (102). As compared to microglia, the chronic response of astroglia provides the opportunity for therapeutic targets. Also, tissue damage and neuronal injuries in chronic conditions may enhance neuroinflammation in glial cells in glaucoma (103). So, adjusting the IOP back to standard or other neuroprotective approaches may help to reduce inflammatory damages (29). Several treatment methods aim to recover this neuronal tissue from neuroinflammatory damages in glaucomatous eyes. This was done by suppressing TNF, the key player in inflammatory and apoptotic signaling at RGC, and axon injuries in *in vivo* models of glaucoma (87, 104). Nevertheless, the exact mechanisms where the TNF signaling provides a shielding or destructing effect must be explained considering the target receptors and cross-stalk among several cascades. Also, to generate neuroinflammation, sTNF is required rather than tmTNF, the primary ligand for TNFR1, the primary receptor for most inflammatory responses (11). The effect of TNF on neuronal survival and inflammatory impact differs according to time-dependent factors (40) and cross interaction of different signaling cascades (105).

By targeting neuroinflammation, drugs known as TAK-242 (resatorvid) has been investigated to deactivate astrocyte *via* inhibition of toll-like receptor 4 signaling pathway, which suppressed the TNF expressions, hence protect against RGC injury in mice with optic nerve injury (106). More studies suggest evidence that neurotoxicity of TNF contributed to the neurodegeneration in glaucomatous models. RGC effects in glaucomatous eyes are determined by a critical balance between diverse signaling cascades. As far as the author is concerned, TNF signaling can cause cell damage and survival. Specific inhibition of cell death signaling and amplification of survival signaling are expected to protect RGCs rather than inhibition of death receptor binding. Because such approaches are not likely to interfere with the survival-promoting signaling elicited by TNF, they might possess superior neuroprotection. Apoptosis signaling caused by TNF in RGCs suggests that neuroprotection targeting this particular marker should include methods to inhibit the caspase production from protecting RGC and improving survival from cytotoxic implications of mitochondrial dysfunction.

Furthermore, several TNFα signaling-associated biomarkers, such as NF-κB and MAPKs aim to govern signals of cell death or survival. More research is needed to investigate the neuroprotective effects of anti-TNF/TNFRs strategies in targeting axonal injury (46). The key glial activation pathways should be recognized for mitigating the glia-associated element of neurodegeneration because glial cells are the primary supplier of TNF (107). Proteomic analysis to investigate the signaling cascades associated with neurodegeneration is among the current strategies in this issue (108). RNA interference technology provides a prevailing implement for determining the functional consequence of newly recognized biomolecules as therapeutic targets using specific siRNAs. This machinery can also be used as an intervention plan in glaucoma, alongside other genomic or drug treatments, to offer neuroprotection.

TNF inhibitors suppress the elevated TNF-mRNA found in the iris and ciliary body experimental autoimmune anterior uveitis (109). Topical TNF inhibitors also effectively neutralize the increased TNF levels in the aqueous humour of idiopathic uveitis patients (110). Of note, elevated TNF levels in the aqueous humour was reported associated with the unsuccessful early trabeculectomy (111). The authors suggest that the cytokine promotes the proliferation of Tenon’s capsule fibroblasts in subconjunctival tissues (111). Indeed, the use of topical TNF inhibitor post-trabeculectomy could alleviate the conjunctival scarring response (112). In the same vein, Leinonen et al. provide a retrospective analysis on the therapeutic potential of systemic TNF inhibitor on Mitomycin C-augmented trabeculectomy with juvenile idiopathic arthritis-related uveitic glaucoma patients (113). Data reported that the individuals treated with TNF inhibitors during Mitomycin C-augmented primary trabeculectomy have better control of IOP than those untreated. This evidently revealed that TNF inhibitors may influence wound healing after trabeculectomy. Unfortunately, to the best of our view, there is no substantial evidence on the effect of TNF inhibition on trabeculectomies in clinical trials have been reported.

In clinical trials of ocular inflammatory diseases, promising findings were reported on individuals with several phenotype uveitis effectively treated with adalimumab and infliximab (114–117). This benefit potentially can be translated as a new therapeutic approach in glaucomatous patients since up-regulated TNF expression has also been implied in glaucoma patients and experimental animal models (54, 55).

Investigating the anti-TNF agents has been growing as the subject of interest in the treatment of neurodegenerative diseases; however, some have shown no beneficial effects. For example, Infliximab, administered intraperitoneally 15 minutes after alkali burn inflicted, is demonstrated to provide significant retinal and corneal protection (118). However, this protection of anti-TNF is shown only in a rapid, IOP-independent pathway to glaucoma, in which TNF, along with other inflammatory cytokines, is generated anteriorly and causes apoptosis of ganglion cells (119). Apart from age as the leading risk factor for glaucoma, the elevation of IOP is the only risk factor that can be modified for glaucoma. Increased IOP is directly associated with RGCs death and TNF, produced by glial cells upon ocular hypertension, implicated as the link in this glaucomatous degeneration (104). Another drug candidate could be Lenercept, a TNFR1-selective protein to neutralize TNF, which emerged from a clinical study conducted in 168 patients of relapsing-remitting multiple sclerosis. This phase II randomized, multi-center, and placebo-controlled trials implying that TNF inhibitors have exacerbated the disease progression with neurologic deficit exhibited more in patients treated with Lenercept as opposed to placebo (120). Besides, several studies using TNF antagonists have reported fail to respond against the disease; instead, some were causing enhanced progression or developed acute side effects with potential adverse events (121). This paradigm could be due to the counterproductive effects of TNF antagonists that spare the physiological functions of TNFRs, including anti-inflammatory, immune regulatory, and regenerative activities (13).

In addition to the abovementioned and despite the success in other diseases, anti-TNF has yet to demonstrate encouraging efficacy in *in vivo* glaucoma models. Although several studies documented contradicting role of TNFR1 and TNFR2 in glaucoma, TNF and its receptor may still serve as attractive targets in glaucoma therapy. For example, RGCs loss induced by TNF is shown to be caused by TNFR2, instead of TNFR1, when TNFR2 knockout mice showed no RGCs degeneration while in TNFR1 knockout mice showed otherwise (122). In another study, TNF was implicated in increasing the survival of RGCs *in vivo via* activation of TNFR1 (123). These observations indicate that while TNFR1 plays detrimental roles in glaucoma, TNFR2 needs to be preserved to counteract damage to the RGC and other retinal cells. This requires selective targeting of the TNF pathway. One of the approaches that can be utilized for anti-TNF in glaucoma is developing pharmacological means with selectivity to TNFR1 or directly to TNFR2. For instance, TNFR1 antagonist only inhibits the function of TNF *via* TNFR1 without inhibiting host defense function *via* TNFR2 as complete inhibition of TNF in neurodegenerative diseases such as glaucoma may be contraindicative. Fischer et al. (124) extensively reviewed the growing knowledge on TNF and its TNFR2 signaling and highlighted the promising results from this TNFR selective approach in various diseases models (124). Although there are no preclinical studies for TNFR selective drugs in glaucoma, multiple observations from other neurodegenerative diseases such as AD indicate that this approach could provide neuroprotective effects while minimizing the side effects of global neutralization of TNF. In the NMDA-induced AD animal model, administration of either ATROSAB, a TNFR1 antagonist, or EHD2-SCTNFR2, a TNFR2 agonist, prevented neurodegeneration and mediated neuroprotection (125). Interestingly, blocking TNFR1 signaling with ATROSAB prevented membrane-bound TNF from activating neuroprotective signaling *via* TNFR2, further explaining the ineffectiveness of anti-TNF as therapeutic in neurodegenerative diseases. In another AD animal model, an anti-TNFR1 Nanobody called TROS showed prevention of cognitive decline by reducing brain inflammation and blocked blood-CSF barrier impairment (126).

## Conclusion and Future Perspectives

Although in this paper, we generally highlighted the neurodegeneration and neuroprotective role in TNFR1 and TNFR2 signaling, respectively, in RGC and glial cells, findings that contradict the idea should not be ignored. The aberrant observations could be due to the lack of in-depth investigations on the role of TNF/TNFR1 and/or TNF/TNFR2 in preclinical glaucoma. The conflicting findings of TNFRs in glaucoma revealed that similar to TNFR1, TNFR2 inclines to project its functions in neurodegeneration. The opposite functions of TNFR1 and TNFR2 in glaucoma could also be due to different tissues in the visual systems than others, suggesting that their activation depends on the type of tissue. Not to forget, we have yet to elucidate the pathogenesis that led to RGCs loss in glaucoma, let alone to figure out the integrative function of a particular receptor. Altogether, from our perspectives, the potential approach to counteract adverse effects of TNF antagonist therapies in the CNS is through exclusive target TNFR1 signaling with localized delivery of inhibitors. Also, since TNFR2 has been shown to antagonize TNFR1 expressions, TNF agonist or gene therapy of which can enhance expression levels of TNFR2, may be beneficial.

By developing therapeutic agents targeting the TNF/TNFRs, it is highly potential to prevent glaucoma in the early stage. However, total inhibition of TNF against its receptors should be totally avoided as it can cause mild to severe side effects. Clearly, the roles of both receptors have yet to be elucidated, and thereby we propose to focus on maintaining the homeostatic function of TNF in the glaucomatous eyes. This approach may allow efficient neutralization of global TNF and avoid integrating unwarranted mechanisms that may interrupt the ocular immune system, including development and maintenance of immune cell populations, self-tolerance, and resistance to non- or infectious agents. TNFRs inhibitors may contribute to undesirable effects of TNF signaling and other inflammatory cascades that would be suppressed globally in the CNS. Nevertheless, in the context of neuroprotection, to further elucidate the roles of TNFRs in glaucoma, these challenges need to be addressed. In conclusion, selective targeting of TNFRs as a potent therapeutic strategy seems a promising avenue for the treatment of glaucoma.

## Author Contributions

LL, SA, MZS, and NAN performed literature search and drafted the manuscript. RK, NAAN, XC, JB, MP and RM supervised and revised the manuscript. All authors contributed to the manuscript and approved the submitted version.

## Funding

This work was supported by grant from Ministry of Higher Education, Malaysia (Grant number: FRGS/1/2020/SKK06/USM/03/2).

## Conflict of Interest

The authors declare that the research was conducted in the absence of any commercial or financial relationships that could be construed as a potential conflict of interest.

## Publisher’s Note

All claims expressed in this article are solely those of the authors and do not necessarily represent those of their affiliated organizations, or those of the publisher, the editors and the reviewers. Any product that may be evaluated in this article, or claim that may be made by its manufacturer, is not guaranteed or endorsed by the publisher.
